# A Rare Occurrence of Amoxicillin-Induced Aseptic Meningitis in a Young Adult: A Case Report

**DOI:** 10.7759/cureus.88133

**Published:** 2025-07-17

**Authors:** Hafiz Tayyab Raza Jilane, Muhammad Awais Ali, Ajith Kurien, Ahmed Elshahawy, Frieder Kleemann

**Affiliations:** 1 Nephrology, Peterborough City Hospital, Peterborough, GBR; 2 Stroke Medicine, Peterborough City Hospital, Peterborough, GBR; 3 Medicine, Peterborough City Hospital, Peterborough, GBR

**Keywords:** amoxicillin, amoxicillin-induced aseptic meningitis, antibiotic-induced meningitis, aseptic meningitis, β-lactam-induced meningitis

## Abstract

Aseptic meningitis (AM) is characterized by clinical manifestations and laboratory findings indicative of meningeal inflammation, without detectable bacterial pathogens in routine cultures. Drug-induced aseptic meningitis (DIAM) is a rare but important etiology. It has been associated with certain drugs, including intravenous immunoglobulin, antiepileptic drugs, monoclonal antibodies, and antibiotics. Amoxicillin-induced aseptic meningitis (AIAM) is an exceptionally rare variant. We present a case of a 25-year-old female who presented with fever and cough for five days. She was prescribed amoxicillin by her general practitioner (GP) and continued the course for five days. Following this, she began to have severe headaches associated with neck stiffness and photophobia. An urgent lumbar puncture (LP) was conducted, and then a combination of intravenous cephalosporins and acyclovir was started empirically, with a differential diagnosis of partially treated meningitis, possibly bacterial in origin. Cerebrospinal fluid (CSF) analysis revealed lymphocytic pleocytosis with low protein and normal glucose levels. Both bacterial and fungal cultures were negative. Subsequently, herpes simplex virus infection was ruled out through serological testing. Hence, the antibiotics and antivirals were stopped, and she was initiated on symptomatic management. The patient recalled a similar episode occurring subsequent to the administration of amoxicillin for a respiratory infection, a detail that was also reflected in her medical records. Considering her clinical presentation, CSF findings, and the previous exposure to amoxicillin coinciding with an episode of meningitis, a multidisciplinary team (MDT) involving Neurology proposed AIAM as a likely diagnosis. Furthermore, her rapid recovery following the cessation of the drug confirmed the final diagnosis of AIAM. This case emphasizes the need for early recognition to avoid unnecessary antimicrobial treatment, as well as recognizing AIAM in a patient, which can prevent recurrent occurrences.

## Introduction

Amoxicillin is a widely prescribed antibiotic used for various bacterial infections in both hospital and outpatient settings. Despite its broad therapeutic applications, it is crucial to recognize its potential adverse effects, including rare yet significant complications such as amoxicillin-induced aseptic meningitis (AIAM).

AIAM is characterized by a temporal association with amoxicillin use [1], cerebrospinal fluid (CSF) leukocytosis (>5 cells/µL), negative bacterial cultures, and symptom resolution upon discontinuation of the drug [2]. Patients with AIAM typically present with symptoms such as headache, fever, nausea, and photophobia. Additional manifestations may include vomiting, muscle pain, chills, and phonophobia. In severe cases, complications such as confusion, speech difficulties, weakness, difficulty walking, and lethargy are reported [2-4].

Although the exact pathophysiology of AIAM remains uncertain, it is suspected to result from hypersensitivity reactions, specifically type IV (delayed-type) hypersensitivity [4-9].

This case report aims to elucidate the clinical presentation, diagnostic evaluation, and management of a patient with AIAM, highlighting the importance of recognizing this condition as a potential differential diagnosis in cases of aseptic meningitis.

## Case presentation

A 25-year-old White British female with no previously known allergies went to the GP after experiencing a week of worsening flu-like symptoms, including fatigue, sore throat, phlegm, and a chesty cough. She was prescribed a seven-day course of amoxicillin (500 mg) to be taken three times daily, considering superimposed bacterial lower respiratory tract infection. However, after completing five days of the antibiotic, she returned to the GP due to continued illness and the development of new, concerning symptoms. These included worsening headaches associated with photophobia and the onset of neck stiffness, pyrexia, tinnitus, and chest pain. In response to her reported chest pain, a D-dimer assay was performed, revealing an elevated level of 3500 ng/mL. The GP initiated rivaroxaban 15 mg twice daily and referred her to the hospital for further evaluation and management.

Upon admission, her vital parameters were within physiological limits, and she was afebrile. The neurological assessment revealed nuchal rigidity with absent Brudzinski and Kernig signs. The initial brain imaging, performed with a non-contrast CT scan, was normal, and the V/Q scan performed later that day was also normal. Hence, the direct oral anticoagulant (DOAC) was stopped. Further brain imaging, including an MRI and MRV (magnetic resonance venography) of the head with contrast, ruled out stroke, space-occupying lesion, and cerebral venous sinus thrombosis.

A lumbar puncture (LP) was subsequently performed, which revealed clear fluid. The CSF analysis demonstrated lymphocytic pleocytosis (white cell count: 110 × 10⁶/L) with a lymphocytic predominance (95%) and normal glucose and protein levels. Given the potential of oral amoxicillin to alter CSF findings, partially treated bacterial meningitis remained the leading differential diagnosis. Empirical treatment with intravenous acyclovir and ceftriaxone was initiated.

Microbiological examination, including direct microscopy and culture, yielded negative results. Analysis of the CSF viral panel revealed no evidence of enteroviruses, herpes simplex virus (HSV) types 1 and 2, varicella-zoster virus, Epstein-Barr virus, or cytomegalovirus. However, she recalled a similar episode from one to two years ago, which also resulted in an admission, during which she underwent a CSF analysis as well. A review of her medical records revealed a similar clinical presentation in 2023, when she was hospitalized following four days of amoxicillin administration for flu-like symptoms accompanying tinnitus and was subsequently diagnosed with viral meningitis. Table 1 presents the LP results of both admissions.

**Table 1:** Comparison of cerebrospinal fluid (CSF) analysis between previous and recent admission (2023 and 2025) HSV: herpes simplex virus; PCR: polymerase chain reaction; g/L: gram per liter; mmol/L: millimole per liter; cells/µL: cells per microliter

Parameter	2023	2025	Reference Range
Time to resolution (days)	11	9	Nil
Protein	0.21	0.28	0.15–0.45 g/L
Glucose	4.2	3.9	2.2–4.0 mmol/L
White cells	50	110	0–5 cells/µL
Red cells	8	92	0–5 cells/µL
Polymorphs	14%	5%	0–3%
Lymphocytes	86%	95%	40–80%
Gram stain and cultures	Negative	Negative	Negative
HSV-1 & 2 PCR	Negative	Negative	Negative
Varicella-zoster (VZV) PCR	Negative	Negative	Negative
Enterovirus PCR	Negative	Negative	Negative
Parechovirus PCR	Negative	Negative	Negative

Upon admission, amoxicillin was immediately discontinued, resulting in the complete resolution of the patient's neck stiffness, pyrexia, and tinnitus within 48 hours. In light of this, a multidisciplinary review, involving the Neurology team, confirmed a diagnosis of AIAM. As a result, all antimicrobial agents were withdrawn. The patient's headache and photophobia gradually improved, with complete resolution achieved by day nine following cessation of amoxicillin. She was discharged in a stable condition, and her medical records were updated to reflect a documented sensitivity to amoxicillin.

## Discussion

AIAM is a rare yet significant adverse drug reaction that poses considerable diagnostic challenges due to its nonspecific clinical and laboratory findings. Drug-induced aseptic meningitis (DIAM) is fundamentally a diagnosis of exclusion, necessitating a comprehensive evaluation to eliminate infectious and other inflammatory etiologies [10]. The precise pathophysiological mechanisms underlying AIAM remain incompletely understood; however, two primary hypotheses have been proposed: (i) direct meningeal irritation and (ii) a delayed-type IV hypersensitivity reaction [7,9,11-13]. Given the well-documented hypersensitivity potential of penicillin, AIAM is predominantly attributed to an immune-mediated response [14,15], potentially involving immune complex deposition, intrathecal cell-mediated or humoral reactions, or chemical arachnoiditis induced by penicillin-binding protein [11,12].

The clinical presentation of AIAM closely mirrors that of infectious meningitis, with symptoms including fever, headache, neck stiffness, and altered mental status. A crucial distinguishing feature is the temporal correlation with drug exposure, with symptom onset typically occurring within three to seven days following amoxicillin administration. CSF analysis frequently reveals lymphocytic pleocytosis, although cases with neutrophil predominance have also been documented [6,7,9,10,13]. Normal glucose concentrations and variably elevated protein levels further substantiate the diagnosis. Negative serological markers, sterile CSF cultures, and unremarkable neuroimaging findings serve as critical differentiating factors from infectious etiologies.

The principal approach to AIAM management involves the immediate discontinuation of the offending agent [16], which generally leads to symptom resolution. Supportive care, including intravenous hydration, analgesia, and corticosteroid therapy in severe cases, may be employed to mitigate symptom severity [2,6,9,17]. Recurrence of aseptic meningitis can occur with re-exposure to the drug [6,9,13,18]. Our patient initially received empirical antimicrobial therapy due to the presumption of infectious meningitis; however, the resolution of symptoms following amoxicillin withdrawal, coupled with negative microbiological and imaging findings, strongly supported the diagnosis of AIAM.

Bihan et al.'s analysis of 329 pharmacological agents implicated in aseptic meningitis identified that antimicrobials accounted for only 11% of cases, with amoxicillin representing a mere 5% of the implicated agents [17]. To date, only 22 cases of AIAM have been reported in the literature [1], underscoring that this is a rare but clinically significant adverse event.

Notably, emerging literature [4,16] indicates a higher incidence of AIAM in males compared to females. The underlying pathophysiological mechanisms contributing to this observed gender disparity remain ambiguous and necessitate further investigation. Given that AIAM is primarily mediated through a delayed-type IV hypersensitivity reaction involving Th1 cells, existing evidence of sex differences in immune responses, specifically the increased prevalence of type IV hypersensitivity reactions of the Th1 subtype and/or a predominance of CD8+ cells in males, may provide a potential explanation for this higher incidence [4,15].

A comprehensive anamnesis, including an in-depth drug history, is crucial in determining the temporal association between amoxicillin use and symptom onset in this patient and plays a key role in reaching the diagnosis. The overall prognosis of AIAM is typically favorable, with full recovery anticipated upon discontinuation of amoxicillin [1]. Increased clinical vigilance is essential to ensure early identification and effective management of this uncommon yet reversible condition.

Evidence suggests that patients who have experienced AIAM may also be at risk for hypersensitivity reactions to cephalosporins [1,6]. Several case reports [6,14] have documented instances of aseptic meningitis following exposure to cephalosporins. While the existing literature includes reports of aseptic meningitis associated with both amoxicillin and cephalosporins individually, no specific cases have been identified in which patients developed aseptic meningitis from cephalosporins after previously experiencing AIAM.

## Conclusions

The diagnosis of AIAM requires a high degree of suspicion. As amoxicillin is being used worldwide, it is important to consider it as a possible cause of meningitis. The time interval between amoxicillin administration and the relative signs and symptoms ranges from two to seven days. A thorough history, particularly regarding previous drug administration, is crucial for every patient with meningitis. AIAM is a diagnosis of exclusion, which requires an extensive evaluation to rule out infectious and other inflammatory etiologies, along with a rapid resolution of the symptoms after drug withdrawal.

## References

[1] Fan Z, He Y, Sun W, Li Z, Ye C, Wang C (2023). Amoxicillin-induced aseptic meningitis: clinical features, diagnosis and management. Eur J Med Res.

[2] Jolles S, Sewell WC, Leighton C (2000). Drug-induced aseptic meningitis. Drug Saf.

[3] Chu L, Eustace M (2018). Recurrent amoxicillin-induced aseptic meningitis. Can J Neurol Sci.

[4] Czerwenka W, Gruenwald C, Conen D (1999). Aseptic meningitis after treatment with amoxicillin. BMJ.

[5] Chandler RE (2019). Increased risk for aseptic meningitis after amoxicillin or amoxicillin-clavulanic acid in males: a signal revealed by subset disproportionality analysis within a global database of suspected adverse drug reactions. Pharmacoepidemiol Drug Saf.

[6] Ponte CD, Unger K (2020). A suspected case of amoxicillin-associated aseptic meningitis. J Pharm Technol.

[7] Romano A, Gaeta F, Arribas Poves MF, Valluzzi RL (2016). Cross-reactivity among beta-lactams. Curr Allergy Asthma Rep.

[8] Nakajima W, Ishida A, Takahashi M, Sato Y, Ito T, Takada G (2007). Aseptic meningitis associated with cephalosporins in an infant with trisomy 21. J Child Neurol.

[9] Sousa D, Raimundo P (2020). Amoxicillin-induced aseptic meningitis. Eur J Case Rep Intern Med.

[10] Shahien R, Vieksler V, Bowirrat A (2010). Amoxicillin-induced aseptic meningoencephalitis. Int J Gen Med.

[11] Scherschinski L, Baudier L, Mono M.L (2021). Amoxicillin-induced meningoencephalitis: case report and review of the literature. Am J Intern Med.

[12] River Y, Averbuch-Heller L, Weinberger M, Meiner Z, Mevorach D, Schlesinger I, Argov Z (1994). Antibiotic induced meningitis. J Neurol Neurosurg Psychiatry.

[13] Alarcón E, Sansosti A, Navarro B, Claver Á, Botey E, Cisteró-Bahima A, Bartra J (2019). Amoxicillin-induced aseptic meningitis: 2 case reports and appraisal of the literature. J Investig Allergol Clin Immunol.

[14] Creel GB, Hurtt M (1995). Cephalosporin-induced recurrent aseptic meningitis. Ann Neurol.

[15] Castagna J, Nosbaum A, Vial T (2018). Drug-induced aseptic meningitis: a possible T-cell-mediated hypersensitivity. J Allergy Clin Immunol.

[16] Mei Mei, P.B.C.S P.B.C.S (2024). Amoxicillin: risk of aseptic meningitis. https://npra.gov.my/index.php/my/component/content/article/454-english/safety-alerts-main/safety-alerts-2024/1527620-amoxicillin-risk-of-aseptic-meningitis.html.

[17] Bihan K, Weiss N, Théophile H, Funck-Brentano C, Lebrun-Vignes B (2019). Drug-induced aseptic meningitis: 329 cases from the French pharmacovigilance database analysis. Br J Clin Pharmacol.

[18] Turk VE, Šimić I, Makar-Aušperger K, Radačić-Aumiler M (2016). Amoxicillin-induced aseptic meningitis: case report and review of published cases. Int J Clin Pharmacol Ther.

